# The Effect of Occupational Engagement on Lifestyle in Adults Living with Chronic Pain: A Systematic Review and Meta-analysis

**DOI:** 10.1155/2022/7082159

**Published:** 2022-06-13

**Authors:** Svetlana Solgaard Nielsen, Søren T. Skou, Anette Enemark Larsen, Alessio Bricca, Jens Søndergaard, Jeanette Reffstrup Christensen

**Affiliations:** ^1^Research Unit for User Perspectives, Department of Public Health, University of Southern Denmark, J. B. Winsloews Vej 9B, 5000 Odense C, Denmark; ^2^The Research Unit PROgrez, Department of Physiotherapy and Occupational Therapy, Næstved-Slagelse-Ringsted Hospitals, Region Zealand, Faelledvej 2C, 4200 Slagelse, Denmark; ^3^Research Unit for Musculoskeletal Function and Physiotherapy, Department of Sports Science and Clinical Biomechanics, University of Southern Denmark, Campusvej 55, 5230 Odense M, Denmark; ^4^Department of Therapy and Midwifery Studies, Faculty of Health Sciences, University College Copenhagen, Sigurdsgade 26, 2200 Copenhagen N, Denmark; ^5^Research Unit for General Practice, Department of Public Health, University of Southern Denmark, J. B. Winsloews Vej 9A, 5000 Odense C, Denmark; ^6^Research Unit for General Practice, Aarhus University, Bartholins Allé 2, 8000 Aarhus, Denmark

## Abstract

**Background:**

Healthy lifestyle is important to decrease health risks in individuals living with chronic pain. From an occupational therapy perspective, human health and lifestyle are linked to occupational engagement in meaningful everyday activities. This study is aimed at investigating the effect of including occupational engagement in chronic pain interventions on lifestyle.

**Methods:**

In this systematic review (PROSPERO reg. CRD42020159279), we included randomized controlled trials (RCTs) on interventions involving occupational engagement (i.e., occupational performance based on involvement, choice, positive meaning, and commitment) and assessing modifiable lifestyle factors: physical activity, body anthropometrics, alcohol consumption, smoking, stress, and sleep. We sought the databases Ovid MEDLINE, Embase, PsycINFO, CINAHL, Cochrane, Scopus, Web of Science, OTseeker, ClinicalTrials.gov, OpenGrey, and the web engine Google Scholar and citations and references of relevant publications. We evaluated methodological quality with the Cochrane risk-of-bias tool 2.0, determined the overall evidence certainty using the GRADE methodology, and performed meta-analysis when two or more trials reported on the outcomes.

**Results:**

Of the 9526 items identified, 286 were full text screened. We included twelve articles with eleven RCTs comprising 995 adults and assessing physical activity, sleep quality, stress, and Body Mass Index. Sufficient data for meta-analysis was only available for physical activity and sleep quality. The meta-analysis suggested a moderate increase in physical activity after behavioral interventions for fibromyalgia and musculoskeletal pain (SMD = 0.69 (0.29; 1.09)) and a small increase in sleep quality up to 6 months after multidisciplinary self-management of fibromyalgia (SMD = 0.35 (95% CI 0.08; 0.61)). The overall certainty of the evidence was deemed low.

**Conclusion:**

Including occupational engagement in chronic pain interventions may increase short-term physical activity and long-term sleep quality. Due to the few available RCTs including occupational engagement in chronic pain treatment for adults living with chronic pain, further high-quality RCTs are needed and will likely change the conclusion.

## 1. Introduction

Chronic pain represents one of the serious threats for human health and well-being, affecting about 11-40% of the population in different countries, being a major cause of disability, and having annual costs related to healthcare expenses and workability loss of about US$635 billion [1]. Moreover, chronic pain is associated with a higher risk of lifestyle challenges such as inactivity, sleep disturbance, unhealthy eating, excessive tobacco smoking, alcohol misuse, and mental stress [2]. An unhealthy lifestyle can further reduce health and increase comorbidity risk, calling for chronic pain interventions to improve modifiable lifestyle factors, such as physical activity, weight, diet, alcohol consumption, tobacco use, stress, and sleep [3]. From the occupational therapy perspective, an important part of a healthy lifestyle is occupational engagement in meaningful and purposeful everyday activities beneficial for lifestyle factors [4]. By engaging people living with chronic pain in occupations that promote a healthier lifestyle, occupational therapy could help initiate lifestyle changes and improve health and well-being.

A recent scoping review underscored the unique role of occupational therapy in chronic pain treatment, highlighting the need for further research on the effectiveness of interventions targeting occupational issues in people living with chronic pain [5]. We also know that occupational therapy can be useful in weight-loss interventions for adults [6], but whether occupational engagement in chronic pain interventions promotes a healthier lifestyle has not been studied yet.

Evidence highlights that rehabilitation should focus on meaning in everyday life rather than on improving function alone [7], as meaning improves health and empowers to health behavior modification for better well-being [8]. Thus, engaging in meaningful and purposeful activities promoting a healthier lifestyle could potentially improve the effectiveness of lifestyle interventions for people living with chronic pain by adding meaningful content.

This systematic review is aimed at investigating whether chronic pain interventions including occupational engagement as “…a sense of involvement, choice, positive meaning and commitment while performing an occupation or activity” [9] would be effective in improving modifiable lifestyle factors, compared to interventions not including occupational engagement or no intervention.

## 2. Methods

This systematic review was reported according to the PRISMA guidelines [10] (protocol registration CRD42020159279, PROSPERO).

### 2.1. Criteria for Inclusion

According to the new classification of chronic pain proposed by the World Health Organization (WHO) in the International Classification of Diseases for mortality and morbidity statistics (ICD-11) [11], randomized controlled trials (RCTs) including adults ≥ 18 years of age with primary pain conditions ≥ 3 months were eligible: widespread chronic pain, chronic primary musculoskeletal pain, chronic primary visceral pain, chronic primary headache, or orofacial pain, including other and unspecified types of chronic primary pain (see Supplementary Materials, Appendix 1A for the included diagnosis codes). Mixed chronic pain diagnoses, including minor representation of cancer-related pain in the cohort along with other eligible chronic pain diagnoses, were allowed for inclusion if the treatment programme was not diagnosis-specific. Peer-reviewed publications in English, German, Italian, Swedish, Norwegian, or Danish were eligible.

The eligible interventions had to target at least one of the primary outcomes and assess changes from baseline to any available follow-up in lifestyle-related parameters considered by the previous evidence modifiable through a chronic pain intervention [3]. The lifestyle-related outcomes were body anthropometrics, e.g., body weight in kilograms (kg; continuous), Body Mass Index (BMI; interval), and waist circumference in centimetres (cm; continuous); physical activity level measured in hours and minutes (continuous) or a number of walking steps (continuous); alcohol consumption in units per week (continuous); cigarettes smoked per week (continuous); self-perceived sleep quality level (ordinal); and self-perceived stress level (ordinal). The lifestyle-related outcomes had to be assessed by validated methods delivering objective measurements (weighing scales, measuring tapes, or pedometers) or self-reports, e.g., the Karolinska Sleep Questionnaire (KSQ), Depression Anxiety, or Stress Scales (DASS). Change in the lifestyle-related outcomes allowed monitoring improvement or decline in health and well-being as recommended by the Initiative on Methods, Measurement, and Pain Assessment in Clinical Trials (IMMPACT) [12].

The eligible interventions also must include an occupational engagement component based on involvement, choice, positive meaning, and commitment, as defined by Creek [9]. This definition corresponded with the understanding of occupational engagement as a core construct in the occupational therapy practice, including elements, such as clients' sense of readiness, interests, wants, needs, choices, active participation, individual capacities, appropriate challenges, and feedback, and linked to the client's environments. Relevant assessment tools and explicit authors' reports on performing meaningful and purposeful daily activities as part of an intervention strategy helped identify eligible interventions. The relevant assessment tools could be those measuring occupational performance, occupational disability, or pain interference with daily activities, e.g., the Canadian Occupational Performance Measure (COPM), or occupational functioning/disability related to self-care, productivity, and/or leisure, e.g., Brief Pain Inventory (BPI), Dallas Pain Questionnaire (DPQ), Fibromyalgia Impact Questionnaire (FIQ), Oswestry Disability Index (ODI), or Pain Disability Index (PDI). The eligible interventions could (a) be delivered by occupational therapists or multidisciplinary teams; (b) have individual, group, or mixed approaches; and/or (c) operate with nonpharmacological treatment methods, alone or in combination with pharmacological treatment. Eligible comparators were interventions not involving occupational engagement as an active component of the impact, i.e., with no planned practising of daily occupations during the intervention period or no intervention.

### 2.2. Criteria for Exclusion

The following pain conditions of nonprimary character according to the ICD-11 [11] were excluded: chronic cancer-related pain, i.e., pain caused by active malignancy or postcancer sequelae; chronic postsurgical and posttraumatic pain; chronic secondary musculoskeletal pain in joints, bones, tendons, muscles, soft tissues, or vertebral column of inflammatory, infectious, autoimmune, or metabolic aetiology; chronic secondary visceral pain; chronic central; and peripheral neuropathic pain, e.g., that caused by stroke or diabetic neuropathy, chronic secondary headache or orofacial pain, or other specified chronic pain. Please see the diagnosis codes excluded in Appendix 1B. Pregnant or postpartum women and particular labor force or social groups receiving treatment specifically related to their work (e.g., athletes, nurses, dentists, musicians, or students), which may narrow the generalisability of the results to those conditions, were considered not eligible for inclusion.

Trials that only assessed physical function with, e.g., Six Minute Walk Test (6MWT) or Time Up to GO (TUG) test, i.e., reported on movement isolated from a meaningful everyday context implied in the occupational engagement definition used in this review [9], were excluded.

### 2.3. Search Method and Study Selection

We searched the databases Ovid MEDLINE, Embase, PsycINFO, CINAHL, Cochrane, Scopus, Web of Science, OTseeker, ClinicalTrials.gov, OpenGrey, and the web engine Google Scholar for relevant publications (first search on November 21-24, 2019). The alerts for the saved literature database searches were monitored regularly until the end of the study inclusion process to detect additional publications. Reference lists of relevant publications were manually searched for eligible articles. We performed the last search repeating the original database-specific search strategy (Appendix 2) on 25 June 2021. PICO format for the clinical question guided the block search process. A librarian specialist in health sciences assisted with adjusting the search terms and strategy.

The first (SSN) and the last authors (JRC) independently screened the identified RCTs for titles and abstracts using a selection form developed for this study (Appendix 3). All RCTs deemed eligible by one of the two authors were checked independently in full text by the same authors. Any disagreement about including individual trials was subject to discussion until consensus. The EndNote X8 software (Clarivate Analytics), released 8 November 2016, and the Covidence systematic review software (Veritas Health Innovation, Melbourne, Australia) available at http://www.covidence.org were used for sourcing and sorting the search results.

### 2.4. Data Extraction

Items recommended by the EQUATOR (Enhancing the QUAlity and Transparency Of health Research) network in the template for intervention description and replication (TIDieR) guided the data extraction [13]. Using a data extraction form developed in the Microsoft Excel software, the first author extracted the following: information on the author(s), year of publication, country of origin, study design, participant characteristics, sample size (total and in groups, at baseline and follow-ups), program title (if any), treatment concepts, comparators, providers of the experimental content, description of the occupational engagement component including the assessment tools used, lifestyle outcomes according to the inclusion and exclusion criteria and their reported assessments at baseline, postintervention, and long-term follow-up. The last author then validated the data extraction. Any disagreements, e.g., interpretation of the occupational engagement component, were solved by consensus.

### 2.5. Methodological Quality Assessment and the Overall Quality of Evidence

The revised version of the Cochrane risk-of-bias tool for randomized trials (RoB 2 tool) informed the risk-of-bias assessment [14]. The authors SSN and AB answered the series of signalling questions grouped in five domains evaluating various aspects of trial design for the outcomes assessed: risk of bias raised from the randomization process, risk of bias due to deviations from the intended interventions, missing outcome data, risk of bias in the measurement of the outcome, and risk of bias in the selection of the reported result. The proposed algorithms for answering the signalling questions guided the risk of bias judgements (“Low,” “Some concerns,” or “High”) within each domain. The overall risk of bias was determined for each RCT based on in-domain assessment. Disagreements between the two authors were solved by discussion until consensus was met. The Cochrane Robvis visualisation tool was used for a tabular summary.

### 2.6. Analysis and Synthesis

We structured the evidence synthesis around the lifestyle outcomes and the effect measures reported in the included trials. We performed a meta-analysis using a random-effect model. For outcome measures of continuous data, a standardized mean difference (SMD) converted to Hedges' *g* to detect corrected (unbiased) effect sizes [15] was calculated when at least two trials reported on the same outcome domain. Grouping pooled result for each lifestyle-related outcome in the meta-analysis was based on the outcome assessments postintervention and at the last available long-term follow-up after the completed intervention. To adjust for differences in the direction of the assessment scales, e.g., when decreasing scores meant not a decline but an improvement in an outcome, we multiplied the mean values of the relevant outcomes by –1 as recommended by Cochrane (para. 9.2.3.2.) [16]. The standard deviations remained herewith unmodified. In insufficient data reports, we calculated the effect estimates from the available data, e.g., frequencies and graphs. SMD (Hedges' *g*) estimates were used to interpret the pooled effect size of including occupational engagement in chronic pain interventions following the general rule of thumb for the interpretation, i.e., that Hedges' *g* ≥ 0.2 represents a small effect, ≥0.5 a moderate effect, and ≥0.8 a large effect.

Measures of consistency (heterogeneity, *I*^2^) were provided for each outcome. The heterogeneity evaluation was guided by the Cochrane group recommendations (para. 9.5.2.) [16], where the inconsistency values were considered on the continuum from 0%, indicating no inconsistency between the results of individual trials, and 100% indicating maximal inconsistency. The method proposed by the Grading of Recommendations Assessment, Development, and Evaluation (GRADE) working group guided the interpretation of the results from the meta-analysis [17]. Evidence from trials not eligible for inclusion in the meta-analysis was summarized narratively. Confidence intervals and the 5% significance level guided the results' interpretation [18]. We evaluated the overall certainty of the evidence using the GRADE approach by grouping the evaluation ratings according to each outcome domain.

## 3. Results

**Figure 1:**
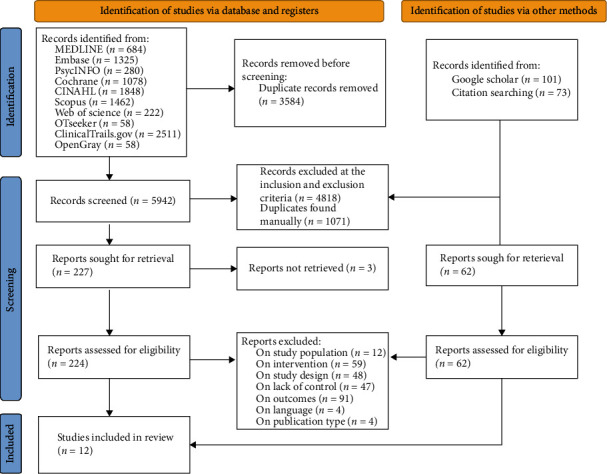
Flowchart for the study.

After excluding duplicates, we screened 6.262 titles and abstracts for eligibility and obtained 286 articles for an independent full-text assessment. Then, we excluded 274 articles, leaving 12 articles reporting on 11 trials for a synthesis (Figure 1).

Reasons for exclusion of articles that initially appeared to meet the inclusion criteria could be an assessment of physical function and not physical activity level [19], promoting occupational engagement in all groups involved [20, 21], or using lifestyle outcomes only to monitor baseline differences between groups [22].

### 3.1. Characteristics of the Included Trials

The twelve articles of the eleven RCTs included 995 adults aged ≥ 18 years (Table 1). Two different papers reported on one RCT [23, 24]. Chronic pain diagnoses represented in the trials were fibromyalgia (*n* = 6) [23–29], low back pain (*n* = 1) [30], pelvic pain (*n* = 1) [31], unspecified musculoskeletal pain (*n* = 2) [32, 33], and minor representation of mixed pain diagnoses, including cancer-related pain along with other (≥ 3) chronic pain diagnoses (*n* = 2) [33, 34]. The included trials reported pain intensity and duration when describing their study samples, but none attempted any pain phenotyping [35].

We observed diversity in intervention approaches, contents, duration, and follow-up time. Although the trials' experimental content generally was client-centered and pain coping-oriented, they used different treatment strategies, such as education [25], behavioral approach [23–25, 27, 29, 32, 33], functional rehabilitation [30], and comprehensive self-management training including didactic information/education, behavior change, and exercise [26, 28, 31]. One RCT compared two treatment strategies and a control group [25]. The comparators were treatment regimens not including occupational engagement such as a brief physiotherapy consultation and advice or exercise prescription [28, 30–33], information/education with no tailored approach [23, 24, 27], waiting list with usual care allowing for variable regimens [26, 29, 34], or a waiting list with no treatment [25]. The intervention descriptions provided in the trials allowed identifying the occupational engagement component and distinguishing it from any alternative. Assessment tools that assisted the identification of the occupational engagement component in the included trials are provided in Appendix 4.

The median duration of the interventions in the sample was 12 weeks (min. 5; max. 12) and six months for long-term follow-up (min. 0; max. 12). Three trials had no other follow-up than that postintervention [27, 28, 31]. One RCT had two follow-up assessments, 6 and 12 months after the ended intervention [24]. Four interventions (36.4%) involved occupational therapists in the multidisciplinary teams of intervention providers (*n* ≥ 2), among physicians, psychologists, physical therapists, occupational therapists, dieticians, or social workers [25, 26, 28, 30]. Four trials had intervention providers representing a single health profession, such as psychologists [34] or physical therapists [31–33]. In three trials, the providers remained unspecified or described as researchers [23, 24, 27, 29]. One of the interventions was delivered online [34].

The following outcomes were assessed in the included trials: physical activity level [23, 24, 27, 30, 32, 33], sleep quality [25, 26, 28, 29], stress [34], and BMI [23, 24]. None of the RCTs assessed waist circumference, alcohol consumption, or smoking outcomes. One RCT assessed multiple (≥2) lifestyle factors of interest in this review [23, 24]. Six RCTs targeted lifestyle explicitly [23, 24, 27, 28, 31, 34].

### 3.2. Risk of Bias

The overall risk of bias was evaluated as low in 9.1% of the trials (*n* = 1), uncertain in 54.5% (*n* = 6), and high in 36.4% (*n* = 4). The results of the critical appraisal of the methodological quality assessment and the weighted summary plot of the methodological quality assessment with the RoB-2 tool are presented in Appendices 5 and 6, respectively.

The randomization procedure was explained in all trials but insufficiently reported in four [25, 27, 28, 34]. The same happened with the reports on allocation concealment in six trials [23–25, 27, 28, 30, 34]. Neither of the included RCTs blinded the participants and intervention providers, which is also difficult when evaluating behavioral interventions. The participants assessed self-reported data in 90.9% of the included trials (*n* = 10), not allowing for blinding the assessors. Two trials used pedometers for walking step assessment [23, 24, 27], while Fontaine & Haaz (2007) used both assessment pedometers and self-reported data.

Six RCTs (54.5%) calculated the sample sizes necessary for their studies [23, 24, 27, 29, 31–33], and one of those failed to reach a sufficient sample size [32]. Three RCTs (27.3%) performed statistical analyses of the data as treated [26, 29, 32] and not the intention-to-treat (ITT) analysis considered the gold standard for randomized controlled design allowing for analysis according to randomization. All three trials reported their losses to follow-up that consisted of 18-31% in the intervention groups and 15-36% in the control groups, where only one trial reporting on physical activity had a higher loss to follow-up among the controls (36% versa max. 18% in the intervention group). One of the included trials had no detailed report on the type of analysis performed [28].

Two trials analyzed participants' adherence to interventions [23, 24, 33], while one analyzed the providers' adherence to the treatment protocol [29]. Five trials reported dropout rates with reasons [27, 30–33].

### 3.3. Effectiveness of Chronic Pain Interventions including Occupational Engagement

**Figure 2:**
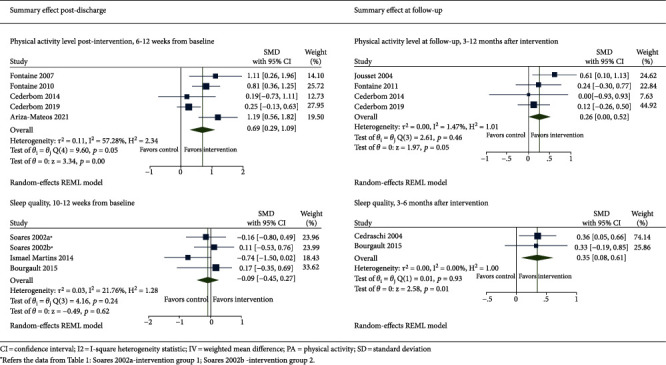
Summary effect of interventions using occupational engagement compared to other or no intervention.

We conducted a meta-analysis on the available data from postintervention and long-term follow-up assessments of physical activity levels assessed in six trials and sleep quality assessed in four trials (Figure 2). Of the two long-term follow-up assessments (at 6 and 12 months) in one of the trials reporting on physical activity level, only the latter was included in the meta-analysis as the most sustainable result [24].

#### 3.3.1. Physical Activity Level

In total, six trials reported on physical activity levels. Our meta-analysis contained effect estimates assessed postintervention from five trials [23, 27, 31–33] and at the long-term follow-up in four trials [24, 30, 32, 33]. All trials compared the experimental treatment with other treatments without the occupational engagement component. The effect estimates favoured intervention. The meta-analysis suggested that including occupational engagement may increase physical activity level compared to other treatments postintervention 6-12 weeks from baseline (SMD = 0.69 (0.29; 1.09)), while the effect was not statistically significant at 3-12-month follow-up after completed intervention (SMD = 0.14 (-0.15; 0.44)). A meta-regression model could help investigate further the substantial level of heterogeneity (*I*^2^ = 57.28%) between the RCTs assessing physical activity postintervention. However, we decided against it since only a few trials were available for comparison, which limited the method's applicability.

All single trials observed increased physical activity in the intervention group, with a significant increase in most [23, 27, 30–32]. However, in adults living with fibromyalgia, physical activity improvement reported postintervention [23] declined to nonsignificant levels at 6-12 months of follow-up [24]. Cederbom et al. observed a similar decline [32].

#### 3.3.2. Sleep Quality

Four trials investigated sleep quality in adults living with fibromyalgia using different treatment approaches [25, 26, 28, 29]. Results deviated from a significant or nonsignificant increase to a decrease in sleep quality were seen in three trials after 10-12-week-long interventions that included occupational engagement [25, 28, 29]. Moreover, Cedraschi et al. had no reports for sleep quality postintervention [26]. The meta-analysis showed no difference in short-term sleep quality (SMD = −0.09 (-0.45; 0.27); *I*^2^ = 21.76%) between the treatment groups and controls.

Based on the data from two RCTs, the meta-analysis found a small effect (SMD = 0.35 (0.08; 0.61)) of multidisciplinary self-management including occupational engagement on sleep quality at 3-6-month follow-up compared to the waiting list receiving usual care with no occupational engagement component [26, 29]. However, the sleep evaluation in the RCT of Cedraschi et al. investigated patient satisfaction with sleep after the treatment received rather than sleep quality like in other trials [26]. The study of Soares and Grossi was excluded from the meta-analysis due to no available long-term follow-up data on sleep quality in the control group. However, the authors reported a significant increase in sleep quality in the behavioral intervention group [25].

#### 3.3.3. Stress

The outcome was represented in only one RCT and, therefore, not eligible for meta-analysis [34]. This RCT compared an online self-management programme for mixed chronic pain diagnoses with a waiting list allowing for usual care and found a significant decrease in stress postintervention and at 14-week follow-up.

#### 3.3.4. BMI

BMI was only assessed in two articles reporting on one RCT and thus not eligible for meta-analysis [23, 24]. This RCT found no significant effect on BMI after a 12-week intervention implementing CBT-informed physical activity in daily life with fibromyalgia compared to fibromyalgia education. No long-term follow-up results (at 6 and 12 months) were reported.

### 3.4. Data Synthesis

Examination applied the GRADE approach demonstrated low to very low level of the overall evidence quality (Table 2). Most included trials had an uncertain or high overall risk of bias, with one trial that had an overall low risk [33]. The risk of bias was the main reason for downgrading the evidence level. Heterogeneity (inconsistency) was also present. Considering the few RCTs per outcome and the low overall evidence quality, we evaluated the total evidence certainty of the effectiveness of including occupational engagement in chronic pain interventions as low.

## 4. Discussion

We hypothesized that interventions including occupational engagement would improve lifestyle in people living with chronic pain and summarized the evidence on the effectiveness of such interventions on the following lifestyle factors: physical activity, sleep, alcohol consumption, smoking, stress, and BMI. Although limited by a small sample and low evidence quality, our study suggested that engagement in daily occupations included as a component in multidisciplinary chronic pain treatment may increase short-term physical activity and slightly increase long-term sleep quality.

### 4.1. Increase in Physical Activity Level

This review detected physical activity increase after behavioral interventions for fibromyalgia and musculoskeletal pain (i.e., low back and pelvic pain) that included occupational engagement, compared to other treatments (e.g., fibromyalgia education and brief physiotherapy advice). Other evidence has pointed out that particularly people living with musculoskeletal and widespread pain can benefit from preventing a sedentary lifestyle [36]. However, it is important to consult the participants of such interventions regarding the expected health benefits. For example, in women with fibromyalgia, lifestyle physical activity improved function, movement fatigue, and physical quality of life but did not reduce pain, pain sensitivity, or pain-related psychological restrain [37]. Nevertheless, universal benefits of physical activity and its ability to give at least a modest effect with few adverse events are well-known [38].

Even a relatively small increase in physical activity may positively impact health, but regular practice is needed. Lower attrition levels in physical activity interventions may improve their effect power [38]. Enjoyable experience with physical activity may enhance self-efficacy towards being physically active, which higher levels were associated with living a less sedentary lifestyle [39, 40]. This review added to the evidence that occupational engagement in self-determined daily activities in self-care, work, and leisure may help accumulate physical activity [41]. Facilitating physical activity through occupational engagement in activities such as gardening and household, performed with moderate intensity, did not increase pain levels [42]. Moreover, it was more pleasurable and motivating than formalised exercise [43].

Surprisingly, we found no eligible trials using holistic mind-body techniques like yoga, Pilates, and tai chi, which anticipate value-based and self-determined participation [44] and are often used in physical activity trials [38]. Most occupational therapy treatment approaches [5, 6, 45, 46] could lead to more physical activity and inspire future interventions including occupational engagement. Occupational therapists may also learn from other healthcare fields and adapt new relevant approaches in occupational therapy practice [47]. However, enhancing physical activity through habitual daily activities implies careful planning and tailoring through, i.e., gradual exposure, energy conservation techniques, and assistive devices to prevent adverse effects [48, 49]. All in all, occupational therapy methods of facilitation physical activity may be useful for people living with chronic pain, but more robust evidence on this topic is needed.

### 4.2. Improvement in Sleep Quality

The slight improvement found in long-term sleep quality up to 6 months after behavioral and educational interventions including occupational engagement should be interpreted carefully because of only two trials included in the meta-analysis. Though the biopsychosocial approach used in the trials seems relevant in managing the complexity of pain and sleep association [50], the treatment benefits were small and appeared only in the longer term.

Sleep is linked to occupational engagement because presleep cognitive arousal strongly predicts sleep quality, and better sleep improves occupational performance the next day [51]. Moreover, the regularity of daily routines and stable circadian rhythms appear essential for sleep quality in humans across different ages [52, 53]. The linkage between occupational balance regarding the number and timing of occupations performed and having appropriate recovery time and good sleep quality has previously been described [54].

Disruptions in the activity-rest cycle represented in many people living with chronic pain increase the frequency of pain flares and pain catastrophising, which, in their turn, negatively impact sleep quality [55]. Poor sleep may then decrease occupational balance the day after, creating a vicious cycle of chronic pain, confirming the tight connection between daily activities and sleep, and making both appropriate targets in chronic pain rehabilitation [56]. However, this review could not tell if this causality also worked vice versa that improved occupational engagement during the day would help improve sleep. Therefore, further trials are needed to investigate how occupational engagement influences sleep in people living with chronic pain.

### 4.3. Decrease in Stress

One RCT reported a significant stress reduction after a web-based intervention targeting various chronic pain conditions through activity pacing, exercise, relaxation, goal setting, and implementation of goal-directed behavior in a population with mixed pain diagnoses, compared to the waiting list. Since stress and pain are linked together due to neural mechanisms in the limbic system [57], we anticipate that any pain management intervention enhancing adaptation to daily life with chronic pain can reduce stress to a certain degree.

Occupational adaptation emerges from occupational engagement in prioritised life situations, which may improve resilience and occupational identity [58]. While the ability to effectively control daily life by using adoptive strategies had previously helped reduce disability risk in older people with elevated cortisol levels [59], we may expect a similar effect in people with other impairments, such as chronic pain. Moreover, occupational adaptation may improve functional and psychosocial health when lowering self-perceived stress and physiological health—by reducing stress biomarkers [60]. However, the quality of the evidence identified in this review was considered very low and did not provide sufficient evidence for a firm conclusion. We urge further research on the topic.

### 4.4. Improvement in BMI

Based on very low-quality evidence, this review could not firmly conclude the effectiveness of including occupational engagement in chronic pain interventions on BMI on basis of. Weight loss interventions are time-consuming and require ongoing monitoring and maintenance [61]. A significant weight loss would unlikely have happened in a relatively brief intervention that does not have BMI as its primary target, like those included in this review.

During the literature search process, we noticed that BMI was often used for baseline comparison between the groups in trials on chronic pain, while it was not an outcome. However, previous research has observed higher frequencies of overweight and obesity in the chronic pain population compared to the general population [62], making the outcome relevant to evaluate in chronic pain trials.

Obesity affects daily living negatively and increases the risks of disability [63]. People living with chronic pain suffering from comorbid obesity have even higher disability risks [64]. Knowing occupational engagement limitations caused by excessive body weight, obese individuals need support promoting participation in meaningful and purposeful activities with attention to evidence-based weight-loss strategies [65, 66]. Occupational therapists can help incorporate obesity impact in chronic pain rehabilitation, promoting sustainable habit changes for weight-loss maintenance in the long term [6]. We suppose that occupational engagement pursuing stable occupational performance patterns would help avoid the yoyo-effect which seems to be characteristic both for ineffective weight-loss attempts and over-/underactivity caused by chronic pain [67]. However, weight-loss interventions for overweight and obese people living with chronic pain need further investigation.

### 4.5. Limitations

Occupational engagement in the chronic pain interventions identified in this review represented one of several treatment components. Involving multiple treatments is characteristic in complex healthcare interventions [68]. Evaluation of treatment doses delivered is necessary when monitoring the dose-response relationship to determine the intervention's effectiveness. Variation in intervention compositions and durations may indicate dose differences in the occupational engagement component delivered, impacting the identified effects. The deliverers' background may also influence clinical reasoning, particularly during the goal work. In the interventions included in the meta-analysis, occupational therapists were mainly involved in the multidisciplinary interventions targeting sleep. Sustainable health behavior changes in sleep quality in the long term may rather reflect the beneficial effect of the multidisciplinary approach than occupational therapy alone [69]. However, occupational therapists may add to the patients' positive mindset by increasing acceptance of living with chronic pain [70]. Anyhow, lack of dose-response clarification may have also limited the conclusions' firmness.

Despite the comprehensive literature search process, we cannot exclude that we missed relevant trials while focusing only on primary chronic pain diagnoses. People living with diagnoses excluded from this review, e.g., osteoarthritis, constitute an essential part of the chronic pain population and share the need for lifestyle improvement [71, 72]. Inclusion of other chronic pain populations might have changed our results, e.g., the effect estimates regarding physical activity level. Two trials had 1-10% of the participants with multiple (≥3) chronic pain diagnoses, inclusive of those related to cancer [33, 34]. However, those two trials differed neither in treatment delivered nor results from the included trials for people without cancer-related pain.

No blinding of the intervention providers and outcome assessors was achievable in any of the included trials; the latter usually due to self-reported data. The subjectivity of evaluations may increase the risk of overestimating the effect favouring the intervention. However, this is very difficult and often impossible due to the nature of occupational interventions.

The effect size in the findings regarding physical activity and sleep can also be biased by lack of ITT [26, 29, 32]. Additionally, there was no differential attrition, but in one trial, loss to follow-up in the controls was higher than that in the intervention group [32]. However, the too few studies included prevented us from investigating such limitations with metaregression and subgroup analyses.

The studies identified in this review needed to have sufficiently explicated study characteristics to allow for their eligibility evaluation, which may also have limited the evidence available for the analysis. Most RCTs in the sample had low methodological quality, and the overall evidence certainty was low. Additionally, none of the included studies attempted chronic pain phenotyping as recommended by the IMMPACT, which would help interpret the differentiated treatment effects and enhance the clinical relevance of the results [35]. The limitations call for a careful interpretation of the results of this review.

### 4.6. Implications for Practice and Research

- Future trials addressing modifiable lifestyle factors could include an occupational engagement component to impact health and well-being
- Occupational therapists can support changing health behavior and healthy lifestyle in people living with chronic pain
- In-detail descriptions of the occupational engagement component and monitoring lifestyle-related outcomes will improve the research transparency and support comprehensive evaluation of the impact on metabolic health in the chronic pain population
- Further research is needed to determine the effectiveness of including occupational engagement in interventions, e.g., targeting physical activity level, sleep quality, stress, and excessive body weight in people living with various chronic pain diagnoses and phenotypes

## 5. Conclusions

This systematic review suggested that occupational engagement in daily activities included as a component in multidisciplinary interventions for chronic pain treatment may increase physical activity in the short term and sleep quality in the long term. However, the overall evidence on the effectiveness of chronic pain interventions including occupational engagement on physical activity level, sleep quality, stress, and BMI in adults with primary chronic pain was low, thereby not allowing for firm conclusions. The impact on smoking and alcohol consumption remained unrevealed. Evidence on the effectiveness of including occupational engagement in chronic pain treatment, alone or in combination with other approaches, is still scarce and demands further rigorously designed investigations.

## Figures and Tables

**Table 1:** Characteristics of the included trials.

Study reference^a^	Participants (diagnoses, *n* randomized/completed, age, gender, pain duration/intensity, and settings)	Intervention and comparators (duration/follow-up, *n* included in the analyses, treatment contents, and providers)	Occupational engagement component explicated, mean (SD), and assessment tools	Lifestyle-related outcomes^b^, mean (SD), and assessment tools
Soares and Grossi, 2002 (RCT), Sweden [25]	Fibromyalgia (100%) *n* = 53/46; ≥18 ≤ 64yr, mean 45(9)yo 100% females Pain duration (y) mean 3.6 (3.3); pain intensity (0-100 scale), mean 85.5(12.6) General practice	10 wk/FU1 (PI) 10 wk/FU2 6mo IG1, *n* = 18/15 at FU2: educational intervention (EI) aimed self-management incl. education, information, and discussions on health-related topics; treatment contract on individual occupational goals; and consultations with experts/OT and PT IG2, *n* = 18/14 at FU2: behavioral intervention (BI) incl. CBT, relaxation, and biofeedback/psychologist and physician CG, *n* = 17: WL (no treatment)	Engaging in active behaviors, CSQ^c^ IG1: intervention informed by individual treatment contract between the patients and the therapists incl. personal expectations, fears, goals, and needs IG2: didactic information and practical training aimed acquisition and development of pain coping skills incl. homework and evaluation	Sleep quality, KSQ^d^ BL, IG1: 3.94 (0.80); IG2: 3.69 (0.83); CG: 3.62 (0.81) FU1, IG1: 3.87 (0.83); IG2: 3.64 (0.91)^∗^; CG: 3.74 (0.80) FU2, IG1: 4.08 (1.04); IG2: 3.21 (1.19)^∗^; CG: NR
Cedraschi et al., 2004 (RCT), Switzerland [26]	Fibromyalgia *n* = 164/129; mean 48.9 (9.7)yo 93% females Pain duration (y) mean 8.9 (5.9); pain intensity (0-105 scale), mean 65.4 (16.9) University clinic	6wk/FU1 (PI) 6wk/FU2 6mo IG, *n* = 84: multidisciplinary self-management programme incl. swimming, relaxation, exercises, ADL impact, and didactic information and discussion/PT, OT, physician, and psychologist CG, *n* = 80: WL (usual care allowed)	Fibromyalgia impact on daily activities, FIQ^e^ Self-management incl. OT sessions aimed difficulties and solutions in ADL monitored through weekly diaries, individual activity planning, and adjusted activity pacing, to minimise fatigue and pain and increase the activity level	Sleep quality, Pott and Silverman questionnaire BL, IG: 2.2 (1.4); CG: 2.1 (1.3) FU1: NR FU2, IG: 2.6 (1.3); CG: 2.1 (1.5)
Jousset et al., 2004 (RCT), France [30]	Low back pain (100%) *n* = 86/83; mean 52.0 (16.7)yo 32.6% females Pain duration (y) mean NS; pain intensity (0-10 scale), mean 4.8(2.2) Regional rehabilitation centers	5wk/FU1 (PI) 5wk/FU2 6mo IG, *n* = 42: multidisciplinary functional restoration program (FRP)/PT, OT, psychologist, and dietician CG, *n* = 41: active individual therapy (AIT) incl. standard functional training and exercise prescription/PT	Pain interference with ADL, DPQ^f^ Pain interference with work/leisure activity, DPQ^g^ OT training for flexibility, endurance and coordination, weightlifting, and work simulation	PA-level (participation in sports/leisure activities), diary BL: NR FU1: NR FU2, IG: 76.2% increase^∗^; CG: 51.2% increase
Fontaine and Haaz, 2007 (pilot RCT), USA [27]	Fibromyalgia (100%) *n* = 48/34; ≥18yr, mean 50.2 (9.1)yo 95.8% females Pain duration (y) mean 7.1 (4.3); pain intensity (0-10 scale), mean 5.7 (5.6) University clinic	12wk/FU (PI) 12 wk IG, *n* = 22/ 14: lifestyle physical activity program (LPA): a CBT-based physical activity promotion program based on Active Living Every Day^g^ incl. self-monitoring, goal setting, and problem-solving aimed integration of moderately intense PA in daily life/NS CG, *n* = 26/20: fibromyalgia education (FME) incl. information on exercise and physical activity but no tailored recommendations/NS	Fibromyalgia impact on daily activities, FIQ^e^ Practicing brisk walking, gardening, mowing the lawn, and using the stairs instead of the elevator of 10 to 30 min. bouts to match PA recommendations	PA-level, pedometer (walking steps) BL, IG: *n* = 2337 (±427); CG: NR FU, IG: *n* = 3970 (±598), 69.8% increase^∗∗^, 71% improvers; CG: NR, 25% improvers
Fontaine et al. 2010 (RCT), USA [23] and Fontaine et al. 2011 (RCT), USA [24]	Fibromyalgia (100%) *n* = 84/73; ≥18yr, mean 47.7 (10.7)yo 95.7% females Pain duration (y) mean 7.6 (6.2); pain intensity (0-100 scale), mean 56.5 (25.3) University clinic	12wk/FU1 (PI) 12wk/FU2 6mo/FU3 12mo IG, *n* = 46/30 at FU2 and FU3: lifestyle physical activity program (LPA): a CBT-based physical activity promotion program based on Active Living Every Day^g^ incl. self-monitoring, goal setting, and problem-solving aimed integration of moderate-intensity PA in daily life/NS researchers CG, *n* = 38/23 at FU2 and FU3: fibromyalgia education (FME) incl. information, question and answer, and social support, with no tailored recommendations (minimal intervention)/NS	Fibromyalgia impact on daily activities, FIQ^e^ Practicing brisk walking, gardening, mowing the lawn, and using the stairs instead of the elevator of 10 to 30 min. bouts to match PA recommendations	BMI, weight (kg) divided by height (m^2^) BL, IG: 31.4 (8.4); CG: 29.8 (6.2) FU1, IG: 31.0 (9.0); CG: 29.9 (6.2) FU2: NR FU3: NR PA-level, pedometer (walking steps) BL, IG: *n* = 4788 (±2135); CG: NR FU1, IG: *n* = 5837 (±1770), 54.0% increased PA^∗∗^; CG: NR/NS FU2, IG: *n* = 4496 (±3228); CG: *n* = 4142 (±2286) FU3, IG: *n* = 4589 (±3190); CG: *n* = 3897 (±2460)
Ruehlman et al., 2012 (RCT), USA [34]	Migraine/headaches, 65.5%; back injury/disease, 60.5%; tension headaches, 41%; OA, 31%; facial/jaw pain, 29%; premenstrual pain, 28%; cluster headache, 16%; pelvic injury/disease, 12%; RA, 7%; cancer, 1% (≤ 3 pain diagnoses per participant) *n* = 305/280; ≥18yr, mean 45yo Pain duration (y) > 2 (89,5%); pain intensity (0-5 scale), mean NS, min. 1.65 (1.58)-max. 3.94 (1.39) 64% females Online	7 wk/FU1 (PI) 7wk/FU2 14wk IG, *n* = 162: online self-management program incl. CBT, interpersonal, and self-management approaches/psychologists CG, *n* = 143: WL, NS usual care (treatment regimens may vary)	Pain interference with daily life, e.g., recreation activity, chores, work, and self-care, PCP-EA^h^ Off-line activities aimed practicing new skills, e.g., exercise and relaxation or/and implementation of personal goal-directed behavior	Stress, DASS^i^ BL, IG: 8.84 (5.53); CG: 7.87 (5.44) FU1, IG: 7.30 (5.01); CG: 7.67 (6.46) FU2, IG: 7.36 (5.21)^∗∗^; CG: 7.64 (5.63)
Cederbom et al., 2014 (RCT, feasibility trial), Sweden [32]	Musculoskeletal pain (100%) *n* = 23/16; ≥65yr, mean 84yo 100% females Pain duration (y) mean 27.5 (21.5); pain intensity (0-100 scale), mean 48.3 (25.7) Municipal primary health care service	12wk/FU1 (PI) 12wk/FU2 3mo IG: *n* = 12 BL/10 at FU1 and 9 at FU2: behavioral medicine intervention added physical therapy principles incl. analysis of individual physical and psychological characteristics, and social and physical environmental factors; environmental impact on the ability to perform everyday activities and difficulties in occupational performance; goal setting; practicing of goal behavior/PT CG: *n* = 11 BL/7 at FU1 and 7 at FU2: brief PA advice/PT	Pain-related disability, CPGQ^j^ Monitoring (activity diary) and modification of duration and intensity of the everyday PA to match PA recommendations	PA-level, the Frändin-Grimby scale BL, IG: 2.4 (0.51); CG: 2.4 (0.52) FU1, IG: 2.7 (0.48)^∗^; CG: 2.6 (0.54) FU2, IG: 2.6 (0.53); CG: 2.6 (0.54)
Ismael Martins et al., 2014 (RCT), Brazil [28]	Fibromyalgia (100%) *n* = 27/27; ≥28 ≤ 67yr, mean 42.5 (9.8)yo 64% females Pain duration (y) mean 4.2 (NS); pain intensity (0-10 scale), mean 6.6 (2.7) University clinic	12wk/FU1 (PI) 12wk IG, *n* = 12: weekly Interdisciplinary Program (WIP) incl. educational activities, physical therapy, stretching, ergonomics, and postural orientations combined with CBT-based strategies and approaches to psychosocial and occupational features/physician, OT, PT, psychologist, and social worker CG, *n* = 15: consultation in pain clinic with walking advice/NS	Fibromyalgia impact on daily activities, FIQ^e^ Integration of a home exercise program, ADL ergonomics, and postural guidance	Sleep quality, PSP^k^ (overnight sleep quality item) BL, IG: 72.2 (8.6); CG: 91.2 (6.4) FU, IG: 92.3 (8.4); CG: 98.3 (7.4)
Bourgault et al., 2015 (RCT), Canada [29]	Fibromyalgia (100%) *n* = 56/56; ≥18yr, mean 48yo 92.9% females Pain duration (y) mean 13.8 (9.9) Pain intensity (0-10 scale), mean 6.5 (1.9) University clinic	12wk/FU1 (PI) 12wk/FU2 3mo (IG and CG)/FU3 6mo/FU4 12mo IG, *n* = 28: multidisciplinary self-management program PASSAGE^m^, incl. tailored exercise therapy and educational/psychological tools for self-management of fibromyalgia/NS CG, *n* = 28: WL, NS usual care (nonpharmacological/pharmacological regimens may vary)	Fibromyalgia impact on daily activities, FIQ^e^ Pain interference with daily activities, BPI^n^ Client-entered approach incl. a patient contract with three personal outcome goals to be met, minimally acceptable changes expected, and an agreement on adherence to the program	Sleep quality, CPSI^l^ (the overall sleep quality score) BL, IG: 2.75 (1.82); CG: 2.89 (2.59) FU1, IG: 4.09 (2.04); CG: 3.72 (2.30) FU2, IG: 4.33 (2.18); CG: 3.57 (2.37) FU3, IG:; CG: NR FU4, IG:; CG: NR
Cederbom et al., 2019 (RCT), Norway [33]	Chronic musculoskeletal pain—orthopedic diseases (88%); rheumatoid arthritis (21%); neurological diseases (20%); diabetes (14%); cancer (10%), mean 3.7 reported diagnoses per participant *n* = 105/105; ≥75yr, mean 85yo 87.6% females Pain duration (y) mean 22.4 (22.5) Pain intensity (0-10 scale), mean 4.5 (1.9) Municipal primary health care service	12wk/FU1 (PI) 12wk/FU2 3mo IG, *n* = 52: behavioral medicine intervention (BMPI) based on integrated behavioral medicine and physical therapy principles incl. tailored goal setting, tailored exercise, and progress monitoring/PT CG, *n* = 53: PA recommendations and advice/PT	Pain interference with daily activities, BPI^n^ Individual exercise doses adjusted to personal goals, e.g., walking indoors/outdoors, safe stairs climbing, or holding balance	PA-level, the Frändin-Grimby scale BL, IG: 2.4 (0.7); CG: 2.4 (0.8) FU1, IG: 2.7 (0.8); CG: 2.5 (0.8) FU2, IG: 2.6 (0.7); CG: 2.5 (0.9)
Ariza-Mateos et al., 2020 (RCT), Spain [31]	Chronic pelvic pain (100%) *n* = 44/44; ≥18 ≤ 65yr; mean 44 (9)yo 100% females Pain duration (y) mean 6.6 (4.9) Pain intensity (0-10 scale), mean 5.9 (1.9) University clinic	6wk/FU (PI) 6wk IG, *n* = 22: client-centered approach to workload-capacity balance incl. didactic information, clarification of time consumption, energy expenditure, attention focus, personal goals, goal work, and evaluation/PT CG, *n* = 22: an information leaflet about chronic pelvic pain, physical activity, fear of movement, false beliefs, active lifestyle, and behavioral advice/PT	Occupational performance and satisfaction, COPM^o^ Client-centered approach incl. determination of painful activities in self-care, productivity, and leisure, and personally adjusted activity exposure plan	PA-level, IPAQ^p^ PA: BL, IG: 1563.65 (918.15); CG: 1220.85 (1040.32) FU^∗^, IG: 2248.53 (1145.21); CG: 1150.55 (573.54)

BL: baseline; BMI: Body Mass Index; CBT: cognitive behavioral therapy; CG: control group; diff.: difference; FU: follow-up; HRQoL: Health-Related Quality of Life; IG: intervention group; incl.: inclusive; min.: minimal; MET: Metabolic Equivalent of Task; mo: month(-s); *n*: number; OA: osteoarthritis; NR: not reported; NS: not specified; OT: occupational therapist; *p*: *p* value; PA: physical activity; PI: postintervention; PT: physical therapist; RA: rheumatoid arthritis; RCT: randomized controlled trial; wk: week(-s); WL: waiting list; y: year(-s); yo: years old. ^∗^*p* < 0.05; ^∗∗^*p* < 0.001. ^a^Author (-s), study design, and country of origin. ^b^Body composition, PA-level, alcohol consumption, smoking, sleep quality, and stress. ^c^CSQ: Coping Strategy Questionnaire; ^d^KSQ: Karolinska Sleep Questionnaire; ^e^FIQ: Fibromyalgia Impact Questionnaire; ^f^DPQ: Dallas Pain Questionnaire. ^g^Described in Blair SN, Active Living Every Day, Human Kinetics, Champaign, IL, 2001; ^h^PCP-EA: Profile of Chronic Pain Extended Assessment; ^i^DASS: Depression Anxiety and Stress Scales; ^j^CPGQ: Chronic Pain Grade Questionnaire; ^k^PSP: Postsleep Protocol; ^l^CPSI: Chronic Pain Sleep Inventory. ^m^Described in Barcellos de Souza J, Charest J, Marchand S. École interactionnelle de fibromyalgie: description et évaluation. Douleur et analgésie. 2007; 20: 213–218; ^n^BPI: Brief Pain Inventory; ^o^COPM: the Canadian Occupational Performance Measure; ^p^IPAQ: the International Physical Activity Questionnaire (Supplementary Materials, see Appendix 4).

**Table 2:** Summary of findings.

Occupational engagement component included in chronic pain treatment of adults compared with other or no treatment				

Patient or population:	Adults with primary chronic pain			
Intervention:	Using occupational engagement			
Comparison:	No occupational engagement component			

Outcomes	Comparator	Anticipated absolute effect (95% CI)	Number of participants (studies)	Quality of the evidence (GRADE) a

(A) Physical activity level, SD units: two different instruments used - (a) 6-point ordinal scale; (b) pedometer-driven walking step count; and c) activity diary. Low scores mean lower physical activity level.	Other treatment (brief advice/ information leaflet/ standard physiotherapy/ fibromyalgia education).	At 6-12-weeks from baseline: SMD 0.69 higher (0.29 to 1.09 higher) g; (b) Observed significant increase in physical activity participation (walking steps) in the intervention group compared to controls.	298 (5)	⊕⊕○○ c d e f (Low)
		At 3-12-months after intervention: (a) SMD 0.26 higher (0.0 to 0.52 higher) g; (b) Observed significant increase in physical activity participation (n registered activities) in the intervention group compared to controls.	257 (4)	⊕⊕○○ c e f (Low)
(B) Sleep quality, SD units: four different instruments used - (a) 9-point ordinal scale^∗^, high scores mean low quality of sleep; (b) 10-point ordinal scale, high scores mean high quality of sleep; (c) 0-5-item Likert scale, high scores mean high quality of sleep; and (d) 30-390-point interval scale, high scores mean high satisfaction with sleep quality/good quality of sleep.	Other treatment (consultation with walking advise) or no treatment (waiting list, usual care allowed).	At 10-12-weeks from baseline: SMD 0.09 lower (0.45 lower to 0.27 higher) g.	300 (4)	⊕⊕○○ c e f (Low)
		At 3-6-months after intervention: (a) SMD 0.35 higher (0.08 lower to 0.61 higher) g; (b) Observed significant increase in sleep quality after a behavioral intervention compared to an educational intervention and controls.	266 (3)	⊕⊕○○ c e f (Low)
(C) Stress level, 4-point ordinal scale used. Lower scores mean stress decrease.	Other treatment (waiting list with non-specified usual care, treatment regimens may vary).	At 14-weeks from baseline: mean 0.93 lower (standard error 0.30), p<0.00.	305 (1)	⊕○○○ c e (Very low)
(D) BMI, calculated from weight (kg) divided by height (m2).	Other treatment (Fibromyalgia education).	At 12-weeks after intervention: mean 1.1 higher (5.3 lower to 2.9 higher).	84 (1)	⊕○○○ b e (Very low)

CI: confidence interval; d: day; MD: mean difference; n: number; SD: standard deviation; SMD: standardized mean difference. Notes: ^∗^in Soares (2002), adjusted for direction; a: quality rated from 1 (very low quality) to 4 (high quality); b: evidence limited by inconsistency; c; evidence limited by imprecision; d: evidence limited by heterogeneity; e: evidence limited by small sample size; f: evidence limited by risk of bias (suspicion of selective reporting bias); g: based on Hedges' g interpretation of effect sizes. GRADE Working Group grades of evidence: high: we are very confident that the true effect lies close to that of the estimate of the effect; moderate: we are moderately confident in the effect estimate/the true effect is likely to be close to the estimate of the effect, but there is a possibility that it is substantially different; low: our confidence in the effect estimate is limited/the true effect may be substantially different from the estimate of the effect; very low: we have very little confidence in the effect estimate/the true effect is likely to be substantially different from the estimate of effect.

## Data Availability

The materials supporting the data extraction, analysis, synthesis, and conclusions in this review can be obtained by contacting the corresponding author.

## Supplementary Materials

Supplementary Materials The supplementary materials in Appendices 1-6 provide information about the included and excluded ICD-11 diagnosis codes, database search strategy, study selection form, assessment tools that guided the occupational engagement component identification, and summaries of methodological assessment of the included trials.
