# Supplementary material for: The Effect of Occupational Engagement on Lifestyle in Adults Living with Chronic Pain: A Systematic Review and Meta-analysis
Source: Occup Ther Int. 2022 Jun 13;2022:7082159. doi: 10.1155/2022/7082159 (PMC9208937; doi:10.1155/2022/7082159)
Supplement: Supplementary Materials — The supplementary materials in Appendices 1-6 provide information about the included and excluded ICD-11 diagnosis codes, database search strategy, study selection form, assessment tools that guided the occupational engagement component identification, and summaries of methodological assessment of the included trials. [file 7082159.f1.zip › Appendix 1. The included and excluded ICD-11 diagnosis codes (1).docx]

**Appendix 1**

The included and excluded ICD-11 diagnosis codes

From: Treede, R. D., Rief, W., Barke, A., Aziz, Q., Bennett, M. I., Benoliel, R., Cohen, M., Evers, S., Finnerup, N. B., First, M. B., Giamberardino, M. A., Kaasa, S., Kosek, E., Lavandʼhomme, P., Nicholas, M., Perrot, S., Scholz, J., Schug, S., Smith, B. H., Svensson, P., (…) Wang, S. J. (2015). A classification of chronic pain for ICD-11. Pain, 156(6), 1003–1007. https://doi.org/10.1097/j.pain.0000000000000160

**A. The included ICD-11 chronic pain diagnosis codes**

MG30.0 Chronic primary pain

MG30.00 Chronic primary visceral pain

MG30.01 Chronic widespread pain

MG30.02 Chronic primary musculoskeletal pain

MG30.03 Chronic primary headache or orofacial pain

8A80.2 Chronic migraine

8A81 Tension-type headache

8A81.0 Infrequent episodic tension-type headache

8A81.1 Frequent episodic tension-type headache

8A81.2 Chronic tension-type headache

8A81.Y Other specified tension-type headache

8A81.Z Tension-type headache, unspecified

8A82 Trigeminal autonomic cephalalgias

DA0F.0 Burning mouth syndrome

8D8A.0 Complex regional pain syndrome

8D8A.00 Complex regional pain syndrome type I

8D8A.01 Complex regional pain syndrome type II

8D8A.0Y Other specified complex regional pain syndrome

8D8A.0Z Complex regional pain syndrome, unspecified

MG30.0Y Other specified chronic primary pain

MG30.0Z Chronic primary pain, unspecified

**B. The excluded ICD-11 chronic pain diagnosis codes**

MG30.1 Chronic cancer related pain

MG30.10 Chronic cancer pain

MG30.11 Chronic post cancer treatment pain

MG30.2 Chronic postsurgical or post traumatic pain

MG30.20 Chronic post traumatic pain (i.e., pain developing or increasing in intensity after a tissue injury)

MG30.21 Chronic postsurgical pain (i.e., developing after a surgical procedure)

MG30.3 Chronic secondary musculoskeletal pain

MG30.30 Chronic secondary musculoskeletal pain from persistent inflammation in joint(s), bone(s), tendon(s), muscle(s), soft tissue(s) or vertebral column

MG30.31 Chronic secondary musculoskeletal pain associated with structural/anatomical changes in joint(s), bone(s) or tendon(s)

MG30.32 Chronic secondary musculoskeletal pain due to disease of the nervous system localized in joint(s), bone(s), tendon(s) or muscle(s) that is related to peripheral or central neurological disorders classified elsewhere

MG30.4 Chronic secondary visceral pain originating from internal organs of the head/neck region, or thoracic, abdominal and pelvic cavities

MG30.5 Chronic neuropathic pain caused by a lesion or disease of the somatosensory nervous system

MG30.50 Chronic central neuropathic pain caused by a lesion or disease of the central somatosensory nervous system

MG30.51 Chronic peripheral neuropathic pain caused by a lesion or disease of the peripheral somatosensory nervous system

MG30.6 Chronic secondary headache or orofacial pain

MG30.60 Chronic secondary orofacial pain comprising orofacial pain disorders that have a clear underlying cause

MG30.61 Chronic dental pain caused by a disorder involving the teeth or associated tissues

MG30.62 Chronic headache or orofacial neuropathic pain

MG30.63 Headache or orofacial pain attributed to chronic secondary temporomandibular disorders

MG30.Y Other specified chronic pain
