# Supplementary material for: The Effect of Occupational Engagement on Lifestyle in Adults Living with Chronic Pain: A Systematic Review and Meta-analysis
Source: Occup Ther Int. 2022 Jun 13;2022:7082159. doi: 10.1155/2022/7082159 (PMC9208937; doi:10.1155/2022/7082159)
Supplement: Supplementary Materials — The supplementary materials in Appendices 1-6 provide information about the included and excluded ICD-11 diagnosis codes, database search strategy, study selection form, assessment tools that guided the occupational engagement component identification, and summaries of methodological assessment of the included trials. [file 7082159.f1.zip › Appendix 2. Database search strategy example (1).docx]

**Appendix 2**

Database search strategy example

**Scopus Elsevier** **search string (23.11.2019)**

TITLE-ABS-KEY ( "chronic pain" OR "persistent pain" OR "long-term pain" OR "chronic widespread pain" OR fibromyalgia OR "complex regional pain syndrome" OR "generalized pain syndrome" OR "chronic headache" OR migraine OR "chronic orofacial pain" OR "chronic facial pain" OR "temporomandibular joint disorder" OR "chronic musculoskeletal pain" OR "chronic back pain" OR "chronic low-back pain" OR "chronic neck pain" OR "chronic cervical pain" OR "chronic whiplash syndrome" OR "chronic shoulder pain" OR "chronic abdominal pain" OR "chronic visceral pain" OR "chronic epigastric pain" OR "chronic bowel pain" OR "irritable bowel syndrome" OR "chronic bladder pain" OR "chronic pelvic pain" OR "chronic chest pain" OR "chronic thoracic pain" OR "chronic thorax pain" OR "burning mouth syndrome" OR "chronic primary pain" OR "chronic unspecified pain" OR "nonspecific chronic pain" OR "non-specific chronic pain" OR "chronic neuropathic pain" OR "chronic neuralgia" OR "chronic facial pain" OR "chronic postherpetic neuralgia" OR "chronic trigeminal neuralgia" OR "chronic radicular pain" OR "chronic radiculopathy" OR "chronic postsurgical neuropathic pain" OR "chronic posttraumatic neuropathic pain" ) AND TITLE-ABS-KEY ( "activities of daily living" OR "daily life activity" OR "daily life activities" OR "meaningful occupation" OR "meaningful occupations" OR "occupation-based intervention" OR "occupation-based interventions" OR "occupation-focused intervention" OR "occupation-focused interventions" OR "occupation-centered intervention" OR "occupation-centered interventions" OR "occupational performance" OR "occupational participation" OR "occupational balance" OR "doing occupation" ) AND TITLE-ABS-KEY ( "healthy diet" OR "healthy food" OR "healthy eating" OR "smoking cessation" OR "alcohol drinking" OR "alcohol consumption" OR "physical fitness" OR "physical activity" OR exercise* OR bmi OR "weight loss" OR "waist circumference" OR stress OR sleep OR habit* OR "daily routine" OR "daily routines" OR "eating habits" OR "healthy lifestyle" )
