# Supplementary material for: The Effect of Occupational Engagement on Lifestyle in Adults Living with Chronic Pain: A Systematic Review and Meta-analysis
Source: Occup Ther Int. 2022 Jun 13;2022:7082159. doi: 10.1155/2022/7082159 (PMC9208937; doi:10.1155/2022/7082159)
Supplement: Supplementary Materials — The supplementary materials in Appendices 1-6 provide information about the included and excluded ICD-11 diagnosis codes, database search strategy, study selection form, assessment tools that guided the occupational engagement component identification, and summaries of methodological assessment of the included trials. [file 7082159.f1.zip › Appendix 3. Selection form (1).docx]

**Appendix 3**

Selection form

| **No** | **Selection criteria** | **Decision** |
| --- | --- | --- |
| 1. | Adults 18-65 yo? | NO = exclude |
| 2. | Pregnant or postpartum women? | YES = exclude |
| 3. | Cancer-related pain or severe psychiatric diagnoses? | YES = exclude |
| 4. | Treatment specified to (either/or):   - Degenerative cartilage or bone structure changes? - Inflammatory diseases? - Central neuro system injury? | Any of the following = exclude |
| 5. | Activity-focused/ activity-based treatment (either/ or):   - Explicitly stated in the intervention description - Occupational therapy tools used? (e.g., COPM; AMPS; OBQ, etc.) - Assessed pain interference with daily activities (PDI; FIQ; ODI; PDQ; PHODA; BPI) - Occupational therapists involved in the study | None of the following = exclude |
| 6. | Outcomes assessing modifiable lifestyle factors   - Physical activity in time or walking steps - Body weight in kg or BMI - Waist circumference - Smoking - Alcohol consumption - Sleep - Stress | NO = exclude |
| 7. | Experimental and evaluation studies? | NO = exclude |
| 8. | Control group (-s)? | NO = exclude |
| 9. | RCT or NRCT? | NO = exclude |
| 10. | Peer-reviewed publication? | NO = exclude |
