# Supplementary material for: The Effect of Occupational Engagement on Lifestyle in Adults Living with Chronic Pain: A Systematic Review and Meta-analysis
Source: Occup Ther Int. 2022 Jun 13;2022:7082159. doi: 10.1155/2022/7082159 (PMC9208937; doi:10.1155/2022/7082159)
Supplement: Supplementary Materials — The supplementary materials in Appendices 1-6 provide information about the included and excluded ICD-11 diagnosis codes, database search strategy, study selection form, assessment tools that guided the occupational engagement component identification, and summaries of methodological assessment of the included trials. [file 7082159.f1.zip › Appendix 4. Assessment tools assisting the identification of the occupational engagement component in the sample studies (1).docx]

**Appendix 4**

Assessment tools assisting the identification of the occupational engagement component in the sample studies*

| **Title and brief description** | **References** |
| --- | --- |
| Modified Brief Pain Inventory (BPI) | |
| BPI measures the extent of pain interference with daily life on 10 items (general activity, mood, walking ability, work, relations with others, sleep, life enjoyment, personal care, recreational activities, and social activities) in the past seven days rated on a 0 (does not interfere) to 10 (completely interferes) scale. The average BPI score expresses the global pain interference. | C. S. Cleeland and K. M. Ryan, “Pain assessment: global use of the Brief Pain Inventory”, *Annals of the Academy of Medicine,* *Singapore,* vol. 23, no. 2, pp.129–138, 1994. |
| Canadian Occupational Performance Measure (COPM) | |
| COPM helps to identify and prioritize personal activity problems related to self-care, productivity, and leisure. Individuals rate the self-determined activities according to the self-perceived importance, performance, and satisfaction with the performance on a 10-point scale. The higher the score, the higher is the importance, performance, and satisfaction. The COPM scores support prioritizing the activities for further goal setting and work. The COPM is valid and sensitive to change as an outcome in pain management. | L. Carpenter, G.A. Baker, and B. Tyldesley, “The use of the Canadian Occupational  Performance Measure as an outcome of a pain management program”, *Canadian Journal of*  *Occupational Therapy*, vol. 68, no. 1, pp. 16–22, 2001. |
| Chronic Pian Grade Questionnaire (CPGQ) | |
| CPGQ measures pain-related disability in 7 items inclusive a total score for pain intensity between 0 and 100 and total score for disability between 0 and 100 based on pain-related disability in 3 specific activities where 0 is ‘no disability at all’ and 10 is ‘impossible to perform the activities’, concerning days with restricted performance of usual activities during the past month. The total CPGQ score classifies pain-related disability according to pain severity and interference (grade I: low disability, low pain intensity; grade II: low disability, high pain intensity; grade III: high disability, moderately limiting pain; and grade IV: high disability, severely limiting pain). | B. H. Smith, K. I. Penny, A. M. Purves et al., “The Chronic Pain Grade questionnaire: validation and reliability in postal research”, *Pain,* vol. 71, no. 2, pp. 141–147, 1997. |
| Coping Strategies Questionnaire (CSQ) | |
| CSQ assesses the extent of engagement in eight different strategies for coping with pain inclusive (a reinterpretation of pain sensations, coping self-statements, ignoring sensations, praying/ hoping, catastrophizing, engaging in active behaviours which divert one’s attention away from pain, and increasing behaviours that reduce pain sensations). The overall perceived coping effectiveness is rated with the items ‘Control over pain’ and ‘Ability to decrease pain’. | A. Rosenstiel and F. Kefee, “The use of coping strategies in chronic low-back pain patients: relationship to patient characteristics and current adjustment”, *Pain,* vol. 17, pp. 33 –44, 1983. |
| Dallas Pain Questionnaire (DPQ) | |
| DPQ measures the pain impact on daily activities, work/ leisure activities, anxiety/ depression, and social interest. | G. F. Lawlis, R. Cuencas, D. Selby et al., “The development of the Dallas Pain Questionnaire: an assessment of the impact of spinal pain on behavior”, *Spine,* vol. 14, pp. 511–516, 1989. |
| Fibromyalgia Impact Questionnaire (FIQ) | |
| FIQ measures the overall impact of fibromyalgia on the everyday. FIQ has had version 1.0 (1991), 2.0 (1997), and 2.1 (current). The first 11 FIQ items assess actual capacities related to domestic activities on a 4-point Likert scale ranging from 0 (always) to 3 (never), while the last 9 FIQ items assess the presence and severity of various symptoms in the past seven days (pain, depression, anxiety, fatigue, morning tiredness, stiffness, job difficulty, work missed, and overall well-being) on a numerical scale ranging from 0 (no symptoms) to 10 (major symptoms). The total FIQ score ranges from 0 to 100. The higher score, the greater is the impact of fibromyalgia. | C. S. Burchardt, S. R. Clark, R. M. Bennett, “The Fibromyalgia Impact Questionnaire: development and validation”, The Journal of Rheumatology, vol. 18, pp. 728–733, 1991.  S. Perrot, D. Dumont, F. Guillemin, J. Pouchot, and J. Coste, “Quality of life in women with fibromyalgia syndrome: validation of the QIF, the French version of the fibromyalgia impact questionnaire”, *The Journal of Rheumatology,* vol. 30, pp. 1054–1059, 2003. |
| Profile of Chronic Pain - Extended Assessment (PCP-EA) | |
| PCP-A assesses pain by 95 items inclusive dysfunctional activity performance in 10 areas of daily living. | L. S. Ruehlman, P. Karoly, C. Newton, and L. Aiken, “The development and preliminary validation of a brief measure of chronic pain impact for use in the general population”, *Pain,* vol. 113, pp. 1–10, 2005.  L. S. Ruehlman, P. Karoly, C. Newton, and L. Aiken, “The development and preliminary validation of the profile of chronic pain: extended assessment battery”, *Pain,* vol. 118, pp. 380–9, 2005. |

*Note.* Briefly described from the data reported in the sample studies
