# Supplementary material for: The Effect of Occupational Engagement on Lifestyle in Adults Living with Chronic Pain: A Systematic Review and Meta-analysis
Source: Occup Ther Int. 2022 Jun 13;2022:7082159. doi: 10.1155/2022/7082159 (PMC9208937; doi:10.1155/2022/7082159)
Supplement: Supplementary Materials — The supplementary materials in Appendices 1-6 provide information about the included and excluded ICD-11 diagnosis codes, database search strategy, study selection form, assessment tools that guided the occupational engagement component identification, and summaries of methodological assessment of the included trials. [file 7082159.f1.zip › Appendix 5. Tabular summary of methodological assessment of the included trials (1).pdf]

Appendix 5

Tabular summary of methodological assessment of the included trials

Study	Risk of bias: D1	D2	D3	D4	D5	Overall
Soares & Grossi, 2002	X	X	X	-	-	X
Cedraschi et al., 2004	+	-	-	-	-	-
Jousset et al., 2004	-	+	+	+	-	-
Fontaine & Haaz, 2007	X	X	-	-	-	X
Fontaine et al., 2010 & 211	-	+	+	-	-	-
Ruehlman et al., 2012	X	-	-	-	-	X
Cederbom et al., 2014	-	-	+	-	X	X
Ismael Martins et al., 2014	X	X	+	-	-	X
Bourgault et al., 2015	-	-	-	-	+	-
Cederbom et al., 2019	+	+	+	-	+	+
Ariza-Mateos et al., 2021	+	+	+	-	-	-

D1: D1
D2: D2
D3: D3
D4: D4
D5: D5

Judgement
X High
- Unclear
+ Low

Note. D1, Risk of bias arising from the randomization process; D2, Risk of bias due to deviations from the intended interventions; D3, Missing outcome data; D4, Risk of bias in measurement of the outcome; and D5, Risk of bias in selection of the reported results
