# Supplementary material for: The Effect of Occupational Engagement on Lifestyle in Adults Living with Chronic Pain: A Systematic Review and Meta-analysis
Source: Occup Ther Int. 2022 Jun 13;2022:7082159. doi: 10.1155/2022/7082159 (PMC9208937; doi:10.1155/2022/7082159)
Supplement: Supplementary Materials — The supplementary materials in Appendices 1-6 provide information about the included and excluded ICD-11 diagnosis codes, database search strategy, study selection form, assessment tools that guided the occupational engagement component identification, and summaries of methodological assessment of the included trials. [file 7082159.f1.zip › Appendix 6. Weighted summary plot of the methodological quality assessment with RoB 2-tool (1).pdf]

Appendix 6

Weighted summary plot of the methodological quality assessment with RoB 2-tool

(The proportion of the performed risk-of-bias judgements within each bias domain, measured by the cumulative weight of available judgement levels)

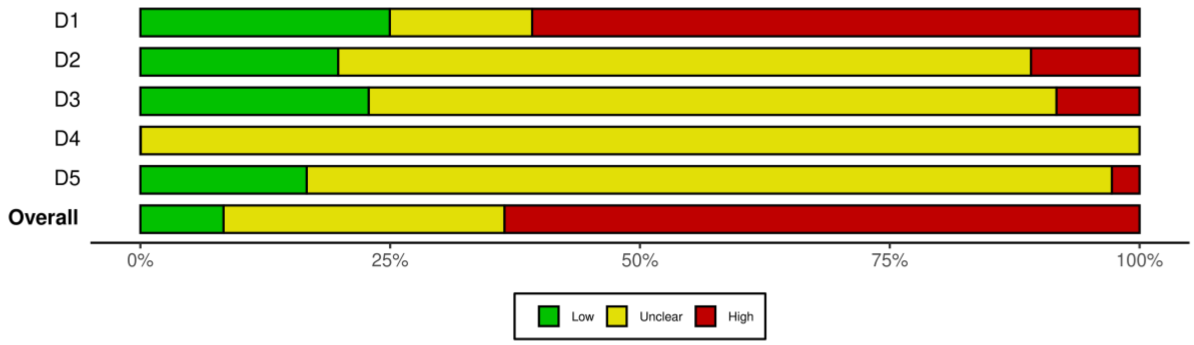

Note. D1, Risk of bias arising from the randomization process; D2, Risk of bias due to deviations from the intended interventions; D3, Missing outcome data; D4, Risk of bias in measurement of the outcome; and D5, Risk of bias in selection of the reported result
